# Bronchial mucosal ablation for bronchial stump closure in right pneumonectomy: a case report

**DOI:** 10.1186/s13256-020-02652-x

**Published:** 2021-02-18

**Authors:** Kimitaka Makidono, Yoshihiro Miyata, Takuhiro Ikeda, Yasuhiro Tsutani, Yuichiro Kai, Kei Kushitani, Yukio Takeshima, Morihito Okada

**Affiliations:** 1 Department of Surgical Oncology, Hiroshima University, 1-2-3-Kasumi, Minami-ku, Hiroshima City, Hiroshima 734-8553 Japan; 2 Department of Pathology, Hiroshima University, 1-2-3-Kasumi, Minami-ku, Hiroshima City, Hiroshima 734-8553 Japan

**Keywords:** Bronchial stump, Bronchial mucosal ablation, Bronchial fistula

## Abstract

**Background:**

Bronchial fistula is a severe complication of pneumonectomy with a high mortality rate. We previously reported a technique for bronchial closure to prevent bronchial fistula in a canine model. We described that mucosal ablation could result in primary wound healing and involve mucosal tight adhesions histologically. In this paper, the pathologic findings of one patient, who underwent autopsy 4 years after surgery, were reviewed.

**Case presentation:**

A 70-year-old Japanese man was diagnosed with malignant pleural mesothelioma and underwent right extra-pleural pneumonectomy. The right main bronchus was cut using a scalpel. When closing the bronchial stump, the bronchial mucosa was ablated by electric cautery and sutured manually using 3-0 absorbable sutures. The bronchial fistula was not found after pneumonectomy. Four years after surgery, the patient died of recurrent malignant pleural mesothelioma and underwent autopsy. Macroscopic evaluation showed tight adhesions and white scars on the bronchial stump. Microscopic findings showed few inflammatory cells and α-smooth muscle actin (α-SMA)-positive cells.

**Conclusions:**

The results from this case suggested that bronchial mucosal ablation leads to robust agglutination of bronchial stump over years. This technique is not only simple but also reliable to prevent bronchial fistula.

## Background

Bronchial fistula is a severe complication following pneumonectomy with a high mortality rate [1–3]. Therefore, a safely effective procedure for bronchial closure is required to prevent bronchial fistula. Several surgical techniques for preventing bronchial fistula have been reported, including coverage using different types of autologous tissues [4, 5] and surgical glues [6] that are commonly used to close bronchial stumps. We previously reported a technique for bronchial closure to prevent bronchial fistula in a canine model [7]. We indicated that mucosal ablation could result in primary wound healing and involve mucosal tight adhesions histologically. In this paper, the pathologic findings of one patient, who underwent autopsy 4 years after surgery, were evaluated.

## Case presentation

**Fig. 1:**
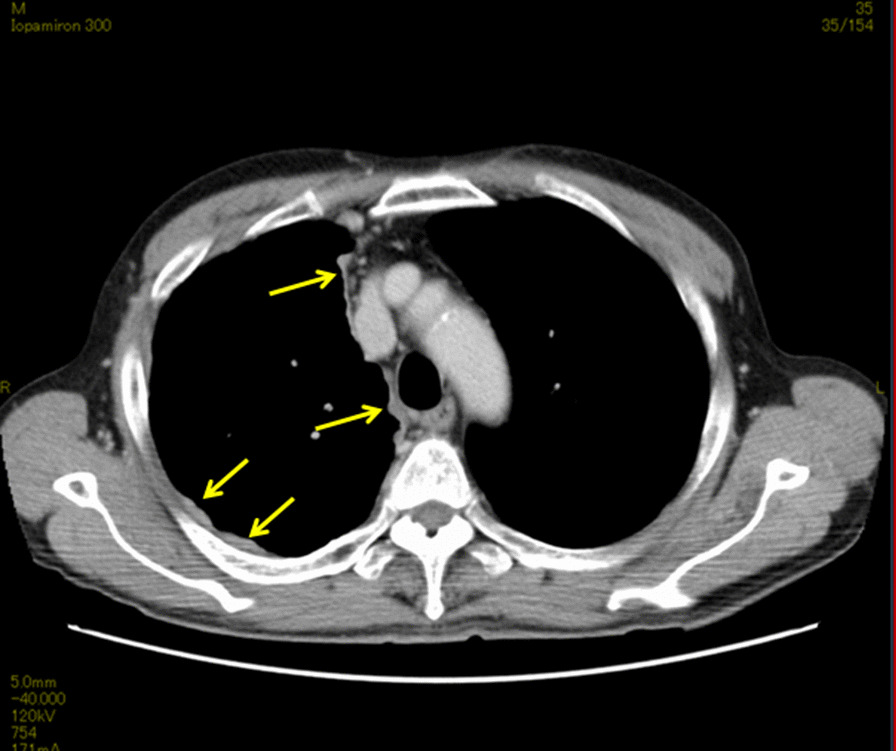
Images showing chest computed tomography (CT) scans. The yellow arrows indicate the diffuse thickened pleura in the right chest cavity. The shape of thickened pleura is also irregular

**Fig. 2:**
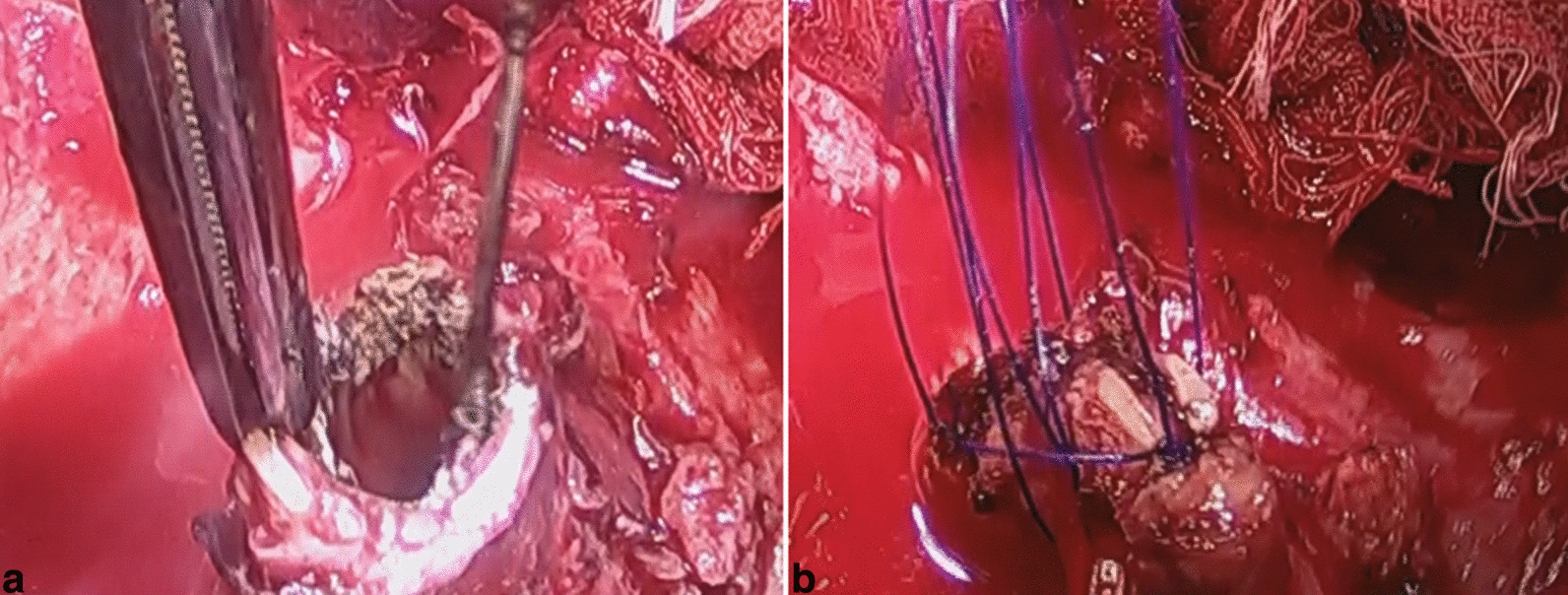
Images showing the surgical procedure. The bronchial mucosa of the stump is ablated using electrocautery (**a**) and sutured manually (**b**)

A 70-year-old Japanese man experienced occasional pain in the right side of the chest and dyspnea. He was diagnosed with malignant pleural mesothelioma and referred to our hospital. Chest computed tomography (CT) revealed diffuse irregular right pleural thickening (Fig. 1). We performed right extra-pleural pneumonectomy after three courses of induction chemotherapy. The right main bronchus was cut using a scalpel. The mucosal surface of the bronchial stump was ablated with electrocautery (Monopolar Cut, blend, at 30 W × 30 W, for 1–2 seconds. Valleylab, ForceTriad™ Energy Platform) with a width of 1 mm (Fig. 2a). The bronchial stump was then sutured manually using 3-0 absorbable monofilament sutures (PDS® II, ETHICON, Inc., Somerville, NJ, USA.). The single ligation suturing technique was used with Sweet’s method (Fig. 2b). No bronchial fistula developed post-operatively.

**Fig. 3:**
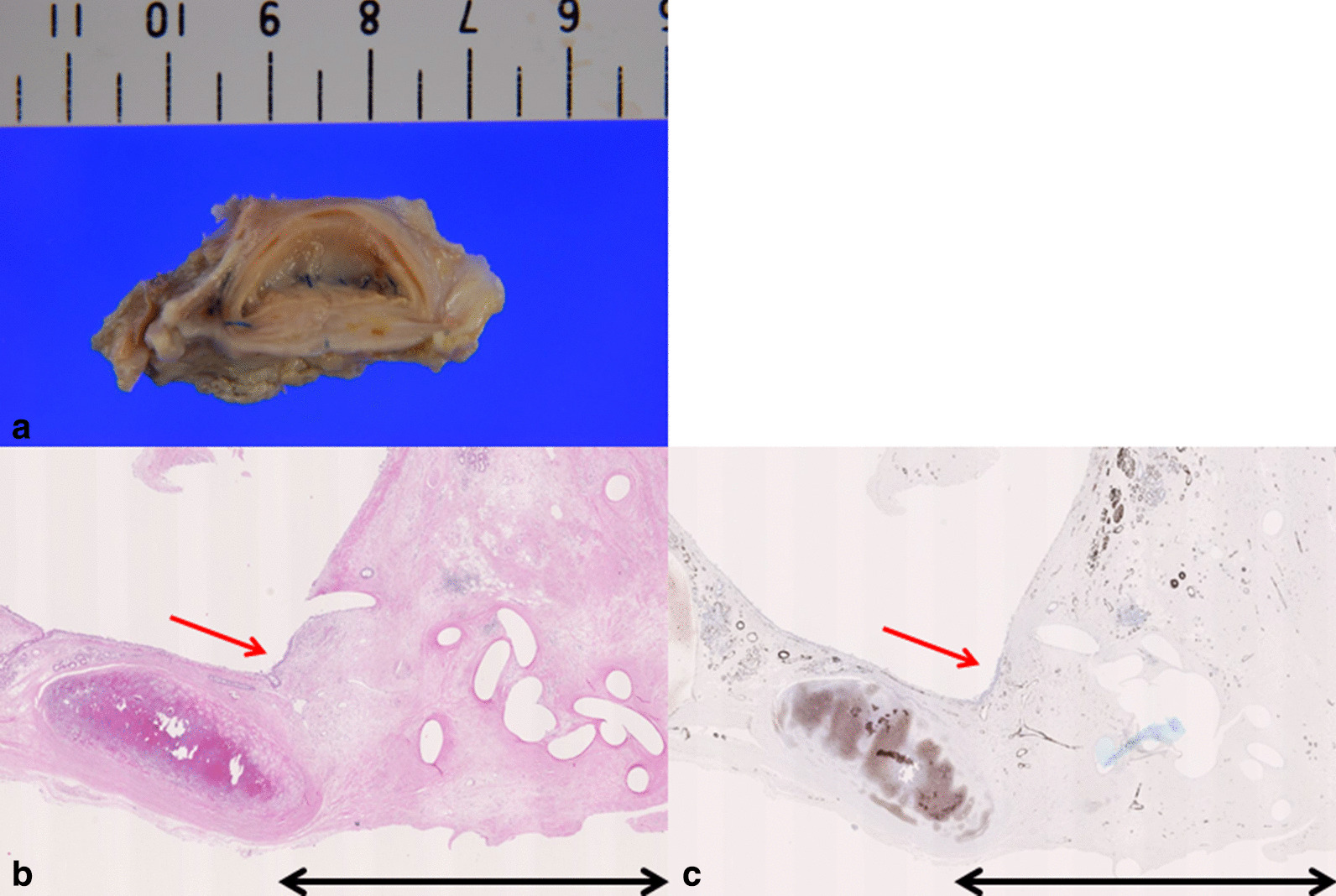
Autopsy findings in a clinical case after pulmonary resection using the bronchial ablation technique. **a** Macroscopic findings. **b** Hematoxylin–eosin staining. × 40 magnification. **c** α-smooth muscle actin (α-SMA) staining. × 40 magnification. The red arrow indicates the innermost layer of the sutured bronchial mucosa, and the black arrow indicates the lesion of mucosal tight adhesion.

Four years after surgery, he died of recurrent malignant pleural mesothelioma, and he underwent autopsy. Macroscopic evaluation showed tight adhesions and white scars on the main bronchial stump and the intervals between the sutured stitches (Fig. 3a). Microscopic findings showed fewer inflammatory cells that were identified around the lesion of mucosal tight adhesion, except for the innermost layer part of the sutured bronchial mucosa (Fig. 3b, c). Furthermore, there were also fewer α-smooth muscle actin (α-SMA)-positive cells in the tight adhesion area (Fig. 3c).

## Discussion and conclusion

Manual suturing techniques, such as the Sweet suture [8] method, have been used for treatment of the bronchial stump for a long time. With the development of surgical devices, mechanical auto-suture techniques are used more recently [9]. However, these techniques could not prevent development bronchial fistula, as seen in conventional techniques [10]. Previous studies in experimental animals indicated that bronchial mucosal adhesion did not occur after both the conventional Sweet manual suture and staple suture techniques. When we ablated the bronchial mucosa before applying the conventional bronchial stump closure technique, a complete union of the mucosa was observed. Our previous method demonstrated that the primary wound healing could provide a robust mucosal agglutination after ablation. It showed that mucosal ablation could trigger the wound healing process and achieve primary closure of the bronchial stump. However, the limitation to our animal study was that we could not observe the long-term outcomes of bronchial ablation.

**Fig. 4:**
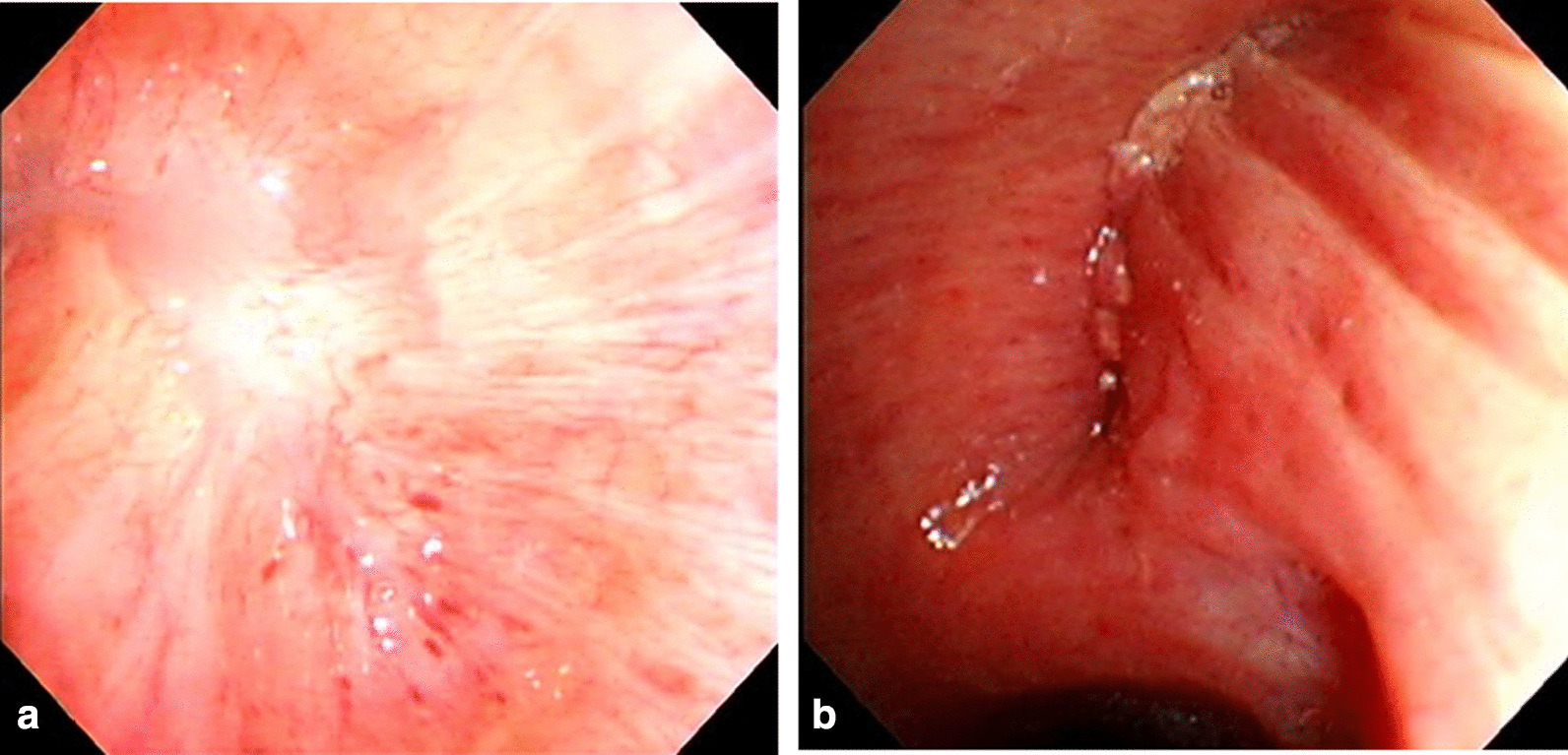
Bronchoscopic findings. **a** Represents the bronchoscopic findings in clinical cases that use bronchial ablation after pulmonary resection. **b** Represents the bronchoscopic findings in clinical cases without ablation after pulmonary resection.

In this case report, we used the bronchial ablation technique in a patient who underwent extra-pleural pneumonectomy with manual suture. A robust mucosal tight adhesion without inflammation was observed at autopsy (Fig. 3). This suggested that in the remodeling phase following mucosal ablation, neither excess inflammation nor granulation was observed, either clinically or histologically. Although we could not observe this case’s inner appearance postoperatively, we used bronchoscopy to examine the mucosal healing process in some cases. It was clear that inner appearance showed tight adhesions and a white scar at the ablation site (Fig. 4a). Figure 4b indicates a non-ablated bronchial stump with manual suture. This inner space was also adhered; however, an obvious mark of the manual suture remained.

Further studies with a higher number of clinical cases are required to confirm our findings. Bronchial mucosal ablation is a potentially beneficial technique for mucosal adhesion. Our clinical findings show that bronchial mucosal ablation resulted in tight adhesion over time.

## Data Availability

The datasets used and/or analyzed during the current study are available from the corresponding author on reasonable request.
